# Dietary Patterns in Pregnancy and Effects on Nutrient Intake in the Mid-South: The Conditions Affecting Neurocognitive Development and Learning in Early Childhood (CANDLE) Study

**DOI:** 10.3390/nu5051511

**Published:** 2013-05-03

**Authors:** Eszter Völgyi, Kecia N. Carroll, Marion E. Hare, Karen Ringwald-Smith, Chandrika Piyathilake, Wonsuk Yoo, Frances A. Tylavsky

**Affiliations:** 1 Department of Preventive Medicine, University of Tennessee Health Science Center, 66 N. Pauline, Memphis, TN 38163, USA; E-Mails: evoelgyi@uthsc.edu (E.V.); mhare@uthsc.edu (M.E.H.); wyoo1@uthsc.edu (W.Y.); 2 Department of Pediatrics, Vanderbilt University Medical Center, 2200 Children’s Way, Nashville, TN 37232, USA; E-Mail: kecia.carroll@vanderbilt.edu; 3 Department of Pediatrics, University of Tennessee Health Science Center, 50 N. Dunlap, Memphis, TN 38103, USA; 4 St. Jude Children’s Research Hospital, 262 Danny Thomas Place, Memphis, TN 38105, USA; E-Mail: karen.smith@stjude.org; 5 Department of Nutrition Sciences, University of Alabama at Birmingham, 326 Webb Nutrition Sciences Building, 1675 University Blvd., AL 35294, USA; E-Mail: piyathic@uab.edu

**Keywords:** nutrient intake, pregnancy, mixed dietary patterns, food frequency questionnaire

## Abstract

Dietary patterns are sensitive to differences across socio-economic strata or cultural habits and may impact programing of diseases in later life. The purpose of this study was to identify distinct dietary patterns during pregnancy in the Mid-South using factor analysis. Furthermore, we aimed to analyze the differences in the food groups and in macro- and micronutrients among the different food patterns. The study was a cross-sectional analysis of 1155 pregnant women (mean age 26.5 ± 5.4 years; 62% African American, 35% Caucasian, 3% Other; and pre-pregnancy BMI 27.6 ± 7.5 kg/m^2^). Using food frequency questionnaire data collected from participants in the Conditions Affecting Neurocognitive Development and Learning in Early Childhood (CANDLE) study between 16 and 28 weeks of gestation, dietary patterns were identified using factor analysis. Three major dietary patterns, namely, Healthy, Processed, and US Southern were identified among pregnant women from the Mid-South. Further analysis of the three main patterns revealed four mixed dietary patterns, *i.e.*, Healthy-Processed, Healthy-US Southern, Processed-US Southern, and overall Mixed. These dietary patterns were different (*p* < 0.001) from each other in almost all the food items, macro- and micro nutrients and aligned across socioeconomic and racial groups. Our study describes unique dietary patterns in the Mid-South, consumed by a cohort of women enrolled in a prospective study examining the association of maternal nutritional factors during pregnancy that are known to affect brain and cognitive development by age 3.

## 1. Introduction

Pregnancy is a time when *in utero* exposures may impact the long term programming for onset of diseases in offspring [1,2,3]. Dietary intake during pregnancy has the potential to influence birth outcomes [4,5] and cognitive development via gene expression [6]. As dietary habits are often cultural and influenced by the food available for consumption, understanding the characteristics of diet within a study population may provide a basis for future interventions to improve lifelong health.

Multivariate statistical methods such as factor analysis has become a well-accepted and popular method [7] to describe dietary patterns in nutritional research. Much of the work has investigated the effects of diet on the risk of adverse outcomes such as colorectal cancer [8,9,10], diabetes and obesity [11,12], and stroke [13]. More recently this approach has been used to characterize diet during pregnancy and relate patterns to nutrient intake, lifestyle and socio-demographic characteristics [14,15,16]. These efforts have provided evidence that dietary patterns may reflect differences in nutrient intake and are sensitive to differences across socio-economic strata or cultural habits. 

The Conditions Affecting Neurocognitive Development and Learning in Early childhood (CANDLE) study is a prospective study that includes a cohort of mother-child dyads. Enrollment for this cohort occurs during the second trimester of pregnancy. The study’s primary aim is to identify factors from *in utero* through early childhood that contribute to cognitive development by age 3. Consistent with life course theory [17] capturing dietary exposure during gestation may be critical in untangling the role of nutrition in the trajectory of early childhood cognitive development. The objectives of this study are to describe a unique process to determine dietary patterns from a food frequency questionnaire (FFQ) that are region specific for the Mid-South, and to examine how these patterns relate to socio-demographic status of the study population, and nutrients (omega 3 fatty acids, folate, pyridoxine, iron, zinc, cobalamin, choline) [18] plausibly linked to neurocognitive development.

## 2. Experimental Section

### 2.1. Study Population

Data from pregnant women who enrolled in the CANDLE study between December 2006 and July 2011 were included in this study. Inclusion criteria included being: a resident of Shelby County Tennessee, able to speak and understand English, aged between 16 and 40 years old, and 16–28 weeks of gestation with a singleton pregnancy.

Exclusion criteria included: an existing chronic disease requiring medication (hypertension, insulin dependent or Type II diabetes mellitus, sickle cell disease or trait, renal disease, hepatitis, lupus erythematous, scleroderma, pulmonary disease, heart disease, human immunodeficiency virus); pregnancy complications including maternal red cell alloimmunization (Rh factor incompatibility permitted); prolapsed or ruptured membranes; oligohydraminios; complete placenta previa; and not intending to deliver at one of four participating hospitals. The study was conducted in accordance with the Helsinki Declaration and was approved and reviewed by the Institutional Review Board of the University of Tennessee Health Science Center. Informed consent was given by all subjects 18 years or older and assent was given by those aged 16–17.9 years with consent provided by their legally authorized representative prior to the assessments. 

### 2.2. Demographic, Lifestyle and Socioeconomic Assessment

Research assistants collected information on family income, participant race, ethnicity, marital status, and parity and household composition through self-administered questionnaires. Pre-pregnancy body height and weight were recorded based on self report of the women. We calculated pre-pregnancy body mass index (BMI) as weight (kg) per height (m^2^).

### 2.3. Dietary Assessment

The Block (2005) food frequency questionnaire (FFQ) was administered during the second trimester by trained research assistants to elicit usual intake of 111 food and beverage groups from the previous three months. Interviewers were trained by registered dietitians and re-certified by a registered dietician based on a taped interview every six months to obtain the frequency of intake and quantity consumed with the aid of standardized food pictures. The FFQ was processed by Nutrition Quest (Berkley, CA, USA) to yield macro and micronutrients, serving size and frequency of intake of the food items. The full Block FFQ has been shown to be a valid and reliable method to describe nutrient intake from diet for groups and rank individuals according to nutrients [19,20,21,22,23].

Of 1503 women who completed the enrollment visit, we excluded respondents who reported implausibly low (<1000) or high (>5000) kcal/day of total energy intake (*n* = 152). Willett [24] reports using an allowable energy range of 500–3500 for non-pregnant, non-lactating women which we adapted for the increased energy needs of pregnancy. Due to technical issues, 196 participants’ FFQ data was unable to be retrieved for determining nutrient intake. The final sample size for this study was 1155.

### 2.4. Food Pattern Determination

Exploratory factor analysis with principal component extraction and varimax rotation method was performed on the frequency of the 111 food and beverage groups to extract the factors that make up distinct dietary patterns. To decide the number of factors to retain, we used the scree plot and the eigenvalues of the principal components, and subjective criteria. We tested solutions for number of models with two to five factors in order to evaluate the interpretability of the dietary patterns. Food groups with a factor loading above 0.30 were considered as the most important contributors to each factor, and were used to identify the dietary patterns. Three factors were identified in our population. These single dietary patterns were termed “Healthy”, “Processed”, and “US-Southern” based on the food groups that loaded for each of the three factors. For each participant, a factor score in the respective single dietary pattern was estimated as a sum of the daily frequency of intake of each food group multiplied by the loading score for the food group. Theoretically the food groups with high daily intake and high factor loading contributed most to the individual’s score in the respective single dietary pattern.

Because the total explained variance of the three single dietary patterns was 15.4%, we explored the use of rank percentiles in order to combine the three major dietary factors, as done elsewhere [16]. After carefully evaluating the factor scores, combined food patterns were created based on the individuals’ rank order in each single factor. Five quintiles were created in each factor based on the individual’s factor scores. (1) Single food patterns (Healthy, Processed, and US Southern dietary patterns) were created if the woman was at least two quintiles higher in one factor than in the other two single dietary factors. (2) A combined dietary pattern was assigned if the individual was at least two quintiles higher in two single dietary patterns than in the third factor. In this way, combined dietary patterns were created as Healthy-Processed (H-P), Healthy-US Southern (H-S), and Processed-US Southern (P-S). (3) If the study participant had less than two quintiles difference between all three single dietary patterns, she was classified with the overall Mixed dietary pattern. As a result, women were grouped into seven mutually exclusive dietary patterns (Healthy, Processed, US Southern, H-P, H-S, P-S, and Mixed), reflecting their primary food choices. Initially, we separated dairy products, salad dressing, and some meat products into low fat and high fat groups. Differential consumption of these foods did not seem to have effect on factor loadings; therefore, we kept low and high fat items in one group.

### 2.5. Statistical Analysis

All continuous data were checked for normality by Shapiro-Wilk’s W test and for homogeneity by Levene’s test before each analysis. Descriptive results are reported as mean ± SE. Body mass index for adults was classified as underweight (<18.5 kg/m^2^), normal (18.5–24.9 kg/m^2^), overweight (25–29.9 kg/m^2^), and obese (>30.0 kg/m^2^). Median test and Kruskal-Wallis ANOVA were used to describe the differences in the demographics and food items among the dietary patterns. Analysis of covariance (ANCOVA) with daily energy intake (kcal/day) as a covariate was used to describe the differences in the macro- and micronutrients among the dietary patterns. The *post-hoc* group differences were evaluated using a Tukey adjustment for multiple comparisons. Multiple regression analysis was performed to describe the explained variance in the macro- and micronutrients by the energy adjusted dietary patterns. All analyses were performed using Statistica v10 (StatSoft Inc., Tulsa, OK, USA) and JMP v9.0 (SAS Institute, Cary, NC, USA). A *p*-value of less than 0.05 was considered statistically significant.

## 3. Results

### 3.1. Description of the Study Participants

Overall, the mean age of the study population was 26.5 ± 5.4 years (range 16 to 40 years). One third of the women in the study population were obese (30%) and one quarter were overweight (24%) before their pregnancies. The women were predominantly African-American (AA) (62%) and 54% of the total sample had a high school degree or less. The number of individuals living in the household ranged from 2 to 11 with the average household size of 4.3 persons, and 37% were single (data not shown).

### 3.2. Dietary Patterns

Factor loadings of the food items for each of the single dietary patterns that had a minimum of 0.30 factor loading are presented in Table 1. The Healthy dietary pattern was characterized by high factor loadings of vegetables, fruits, non-fried fish and chicken, and water. The Processed dietary pattern represents those who consume primarily processed meat, fast food items (items typically obtained from Western-style fast food restaurants), snacks, sweets, and soft drinks. The US Southern pattern was characterized by the typical US Southern foods such as eggs, cooked cereals, peaches, corn, fried fish, beans, greens, cabbage, sweet potatoes, liver, pig’s feet, neck bones oxtails, and tongue, pork, and real fruit juices.

Our statistical approach resulted in 135 (12%) women categorized as Healthy; 98 (8%) as Processed; 120 (10%) as US Southern; 136 (12%) as P-S; 123 (11%) as H-P; 98 (8%) as H-S; and 445 (39%) as Mixed (Table 2). The seven dietary patterns reflect differences in the daily frequency of intakes for the 62 food and beverage groups that loaded from factor analyses from (*p* < 0.001). There were no differences in foods that were commonly consumed by all participants such as cold cereal and milk on cereal and for foods consumed relatively infrequently by only a few participants (menudo, oysters and diet shakes). Overall the daily frequency of the food and beverage items/groups reflects the influence of the respective single pattern when combining the single diet pattern scores. The overall Mixed dietary pattern obtained from the Healthy, Processed and US Southern reflect foods from all of the single, *i.e.*, H-P pattern represents primarily healthy foods but contains some pertinent processed foods. The largest group of mixed patterns contained pertinent food items from all single diet patterns. There were no food items that distinguished it from the three single and three combined dietary patterns. The contribution of the food items from the single diet patterns’ (Healthy, US Southern, Processed) to the mixed groups (H-S, H-P, S-P) could be considered as positive influences (fruits, nuts, seeds, vegetables) or negative influences (salty snacks, higher fat items) on the nutrient density of the diets. For example, in the H-S group there was a slightly higher intake of yogurt than those who reported a “pure US Southern diet”, which has positive influences on calcium and the B complex nutrients. On the other hand, in the H-S group, there was a mildly negative influence of salty snacks that could increase a lower nutrient dense diet. Of the 62 food groups that loaded on the three main groups, there were increases in daily frequency for 39 “healthy” foods that would boost the nutrient density of the diet (62%) and a negative influence on nutrient density from 6 (10%) “less healthy foods” for those in the H-S pattern (Table 2). In contrast the mixed groups that had combined with the Processed group, showed a 25% increase in “negative nutrient dense foods” and 32% increase in nutrient dense foods for the H-P pattern and only a 20% increase in positive nutrient dense foods for those consuming a P-S pattern. From regression analyses the energy adjusted dietary patterns explained 90% of the variance in total fat intake, 84% for protein, 89% for carbohydrate. Regression analyses also showed the energy adjusted dietary patterns explained 62% of the variance for omega3, 65% for sugar, 62% for fiber, 75% for iron, 76% for zinc, 65% for B_6_, 51% for B_12_, 59% for folate, 78% for thiamine, 76% for niacin, 74% for riboflavin, 73% for total choline and 78% for free choline. The explained variances are based on the *R*^2^ of the regression model.

**Table 1:** Factor loadings of food and beverage items/groups in the three main factors.

Food Item (Variance Explained)	Healthy (5.8%)	Processed (5.1%)	US Southern (4.4%)
*BREAKFAST ITEMS*			
Eggs			**0.344**
Breakfast sausage including in sandwiches/biscuits		**0.301**	**0.311**
Bacon			**0.367**
Cooked cereals (oatmeal, grits, cream of wheat)			**0.320**
Breakfast or cereal bars	**0.308**		
*DAIRY*			
Yogurt, including frozen	**0.434**		
Cheese, sliced or spreads		**0.332**	
Milk as a beverage	**0.303**		
*FRUITS*			
Banana	**0.334**		
Apples or pears	**0.355**		
Peaches or nectarines, fresh			**0.352**
Canned fruit			**0.374**
Strawberries or other berries in season	**0.383**		
*VEGETABLES*			
Broccoli	**0.331**		**0.312**
Carrots or mixed vegetables with carrots	**0.471**		
Corn			**0.316**
Green beans or green peas			**0.407**
Spinach, cooked	**0.370**		
Greens (collards, turnip, or mustard)			**0.517**
Sweet potatoes, yams			**0.360**
Fried potatoes (French fries, home fries, hash browns)		**0.563**	
Cole slaw, cabbage, Chinese cabbage			**0.444**
Green salad, lettuce salad	**0.600**		
Tomatoes, raw	**0.524**		
Other vegetables ( squash, cauliflower, okra, peppers)	**0.547**		
Pinto, black or baked beans, chili with beans	**0.304**		
Vegetable, vegetable-beef or tomato soup	**0.337**		
*BREADS*			
Sandwich buns		**0.469**	
Bagels, English muffins, dinner rolls	**0.339**		
Cornbread, corn muffins, hush puppies			**0.380**
Sliced bread (white, dark, whole wheat)		**0.310**	
*CONDIMENTS*			
Salad dressing, regular or low fat	**0.541**		
Mayonnaise, sandwich breads		**0.372**	
Ketchup, salsa or chili peppers		**0.450**	
Mustard, barbecue sauce, soy sauce, gravy *etc.*		**0.322**	
*SWEETS AND SNACKS*			
Donuts		**0.304**	
Cake, snack cakes, cupcakes, Ho-Hos, pastries		**0.437**	
Cookies		**0.407**	
Chocolate candy		**0.392**	
Candy, hard, skittles, starburst *etc.*		**0.358**	
Snack chips like potato chips, tortilla chips, Fritos, Doritos, popcorn		**0.552**	
*MEAT, FISH, POULTRY, MEAT SUBSTITUTES*			
Pizza		**0.328**	
Meat substitutes (veggie burgers, chicken, hot dogs or lunch meats)	**0.346**		
Hamburgers or cheese burgers		**0.504**	
Hot dogs or sausage (Polish, Italian or chorizo)		**0.342**	
Lunch meats (turkey or regular)		**0.393**	
Tacos, burritos, enchiladas, tamales with meat or chicken		**0.306**	
Ribs, spareribs			**0.373**
Liver (chicken livers or liverwurst)			**0.319**
Pigs feet, neck bones, oxtails, tongue			**0.419**
Beef or pork dishes (beef stew, pot pie, hamburger helper)			**0.319**
Fried chicken (nuggets, wings or patties)		**0.497**	
Roasted or broiled chicken or turkey	**0.354**		
Fried fish or fish sandwich			**0.378**
Fish not fried	**0.435**		
Peanut Butter	**0.378**		
Peanuts, sunflower seeds, or other nuts and seeds	**0.348**		
*BEVERAGES*			
100% orange or grapefruit juice			**0.310**
Hi-C, Cranberry Juice Cocktail, Hawaiian Punch, Tang		**0.309**	
Kool-aid, lemonade, sports drinks, or fruit flavored drinks		**0.356**	
Soft drinks (Coke, Sprite, Orange) regular or diet		**0.377**	
Water tap or bottled	**0.320**		

Only those food items are presented that had a minimum of 0.30 factor loading. In a supplementary table, all food and beverage items’ factor loadings are available for each main factor (Supplementary Table S1).

**Table 2:** Average monthly frequency of intake of food groups in the seven dietary patterns^1^.

	Healthy	Processed	Southern	H-P	H-S	P-S	Mixed
Food Groups	(*n* = 135)	(*n* = 98)	(*n* = 120)	(*n* = 123)	(*n* = 98)	(*n* = 136)	(*n* = 445)
BREAKFAST ITEMS							
Eggs	4.4 ± 0.6	4.6 ± 0.7	7.2 ± 0.6	4.3 ± 0.6	10 ± 0.7	7.9 ± 0.6	5.7 ± 0.3
Breakfast sausage including in sandwiches/biscuits	0.8 ± 0.5	4.9 ± 0.6	5.8 ± 0.5	3.0 ± 0.5	3.3 ± 0.6	9.7 ± 0.5	4.0 ± 0.3
Bacon	1.7 ± 0.6	5.5 ± 0.6	7.4 ± 0.6	2.9 ± 0.6	5.2 ± 0.6	8.5 ± 0.5	4.5 ± 0.3
Cooked cereals (oatmeal, grits, cream of wheat)	5.2 ± 0.7	2.8 ± 0.8	7.5 ± 0.7	3.1 ± 0.7	11.0 ± 0.8	5.2 ± 0.7	5.1 ± 0.4
Breakfast or cereal bars	6.6 ± 0.6	2.4 ± 0.7	2.8 ± 0.6	7.0 ± 0.6	4.9 ± 0.7	1.8 ± 0.6	4.6 ± 0.3
DAIRY							
Yogurt, including frozen	11.1 ± 0.7	2.0 ± 0.8	3.5 ± 0.7	7.2 ± 0.7	9.6 ± 0.8	2.8 ± 0.7	5.6 ± 0.4
Cheese, sliced or spreads	16.3 ± 0.8	18.9 ± 1.0	10.7 ± 0.9	21.2 ± 0.9	13.9 ± 1.0	15.5 ± 0.8	15.5 ± 0.5
Milk as a beverage	16.7 ± 1.0	6.9 ± 1.2	8.7 ± 1.1	14.7 ± 1.1	14.7 ± 1.2	7.2 ± 1.0	12.0 ± 0.6
FRUITS							
Banana	9.7 ± 0.7	3.7 ± 0.8	6.2 ± 0.8	6.8 ± 0.7	11.2 ± 0.8	5.4 ± 0.7	6.6 ± 0.4
Apples or pears	11.1 ± 0.7	3.2 ± 0.8	7.4 ± 0.8	6.2 ± 0.7	13.7 ± 0.8	7.2 ± 0.7	7.3 ± 0.4
Canned fruit	2.5 ± 0.3	5.0 ± 0.8	7.9 ± 0.8	4.3 ± 0.6	8.8 ± 1.0	7.7 ± 0.8	6.1 ± 0.4
Peaches or nectarines, fresh	2.3 ± 0.5	1.3 ± 0.6	5.0 ± 0.5	1.9 ± 0.5	7.4 ± 0.6	3.5 ± 0.5	3.2 ± 0.3
Strawberries or other berries in season	9.2 ± 0.6	2.8 ± 0.7	4.7 ± 0.7	6.3 ± 0.7	9.8 ± 0.7	3.9 ± 0.6	5.9 ± 0.3
VEGETABLES							
Broccoli	4.3 ± 0.5	1.9 ± 0.6	4.2 ± 0.5	3.8 ± 0.5	8.6 ± 0.6	3.7 ± 0.5	4.1 ± 0.3
Carrots or mixed vegetables with carrots	6.6 ± 0.5	1.3 ± 0.6	2.4 ± 0.5	4.5 ± 0.5	7.5 ± 0.6	2.0 ± 0.5	3.7 ± 0.3
Corn	3.4 ± 0.5	3.2 ± 0.5	4.9 ± 0.5	5.0 ± 0.5	6.8 ± 0.5	4.9 ± 0.5	5.0 ± 0.3
Green beans or green peas	5.1 ± 0.5	3.6 ± 0.6	6.5 ± 0.5	5.5 ± 0.5	10.2 ± 0.6	5.6 ± 0.5	6.3 ± 0.3
Spinach, cooked	2.6 ± 0.3	0.6 ± 0.4	1.3 ± 0.3	1.9 ± 0.3	4.5 ± 0.4	0.6 ± 0.3	1.2 ± 0.2
Greens (collards, turnip, or mustard)	0.6 ± 0.3	1.0 ± 0.4	3.3 ± 0.4	0.9 ± 0.3	4.8 ± 0.4	3.1 ± 0.3	2.3 ± 0.2
Sweet potatoes, yams	1.4 ± 0.2	0.8 ± 0.3	1.8 ± 0.2	0.8 ± 0.2	3.3 ± 0.3	1.4 ± 0.2	1.3 ± 0.1
Fried potatoes (French fries, home fries, hash browns)	3.0 ± 0.6	13.1 ± 0.7	4.6 ± 0.6	8.9 ± 0.6	4.0 ± 0.7	11.7 ± 0.6	7.6 ± 0.3
Cole slaw, cabbage, Chinese cabbage	1.00 ± 0.2	0.7 ± 0.3	2.0 ± 0.3	0.8 ± 0.3	4.1 ± 0.3	1.7 ± 0.2	1.6 ± 0.1
Green salad, lettuce salad	13.20 ± 0.6	3.9 ± 0.7	5.0 ± 0.6	10.4 ± 0.6	12.1 ± 0.7	3.5 ± 0.6	7.6 ± 0.3
Tomatoes, raw	10.40 ± 0.6	2.6 ± 0.7	2.2 ± 0.6	9.7 ± 0.6	7.5 ± 0.7	2.2 ± 0.6	5.3 ± 0.3
Other vegetables (squash, cauliflower, okra, peppers)	7.00 ± 0.4	0.7 ± 0.5	1.4 ± 0.5	5.1 ± 0.5	8.4 ± 0.5	1.0 ± 0.4	2.7 ± 0.2
Pinto, black or baked beans, chili with beans	3.30 ± 0.3	1.6 ± 0.3	1.3 ± 0.3	2.9 ± 0.3	2.3 ± 0.3	1.6 ± 0.3	2.2 ± 0.1
Vegetable, vegetable-beef or tomato soup	2.1 ± 0.2	0.6 ± 0.3	1.2 ± 0.2	1.2 ± 0.2	2.3 ± 0.3	0.7 ± 0.2	1.5 ± 0.1
BREADS							
Sandwich buns	3.0 ± 0.5	9.4 ± 0.5	3.1 ± 0.5	7.1 ± 0.5	2.5 ± 0.5	6.6 ± 0.5	5.5 ± 0.3
Bagels, English muffins, dinner rolls	4.8 ± 0.4	2.2 ± 0.5	1.6 ± 0.5	6.1 ± 0.5	3.6 ± 0.5	1.9 ± 0.4	3.7 ± 0.2
Cornbread, corn muffins, hush puppies	0.6 ± 0.3	2.0 ± 0.4	2.1 ± 0.3	1.1 ± 0.3	2.3 ± 0.4	3.8 ± 0.3	2.5 ± 0.2
Sliced bread (white, dark, whole wheat)	12.6 ± 0.8	15.8 ± 1.0	8.3 ± 0.9	16.2 ± 0.9	11.1 ± 1	11.2 ± 0.8	12.2 ± 0.5
CONDIMENTS							
Salad dressing, regular or low fat	12.1 ± 0.6	4.9 ± 0.7	5.2 ± 0.7	10.7 ± 0.7	11.9 ± 0.7	4.4 ± 0.6	8.5 ± 0.3
Mayonnaise, sandwich breads	3.3 ± 0.6	9.8 ± 0.7	3.5 ± 0.7	6.9 ± 0.6	4 ± 0.7	8.7 ± 0.6	6.1 ± 0.3
Ketchup, salsa or chili peppers	6.3 ± 0.6	11.9 ± 0.7	3.4 ± 0.7	11 ± 0.6	3.7 ± 0.7	9 ± 0.6	6.7 ± 0.3
Mustard, barbecue sauce, soy sauce, gravy, *etc.*	6.3 ± 0.6	8.5 ± 0.7	3.3 ± 0.6	9.3 ± 0.6	5.5 ± 0.7	6.4 ± 0.6	6.0 ± 0.3
SWEETS AND SNACKS							
Donuts	0.7 ± 0.2	1.2 ± 0.2	0.4 ± 0.2	1.6 ± 0.2	0.4 ± 0.2	1.5 ± 0.2	1.1 ± 0.1
Cake, snack cakes, cupcakes, Ho-Hos, pastries	1.8 ± 0.5	8.2 ± 0.5	1.5 ± 0.5	4.6 ± 0.5	1.3 ± 0.5	5.3 ± 0.5	2.9 ± 0.3
Cookies	3.6 ± 0.5	5.5 ± 0.5	1.5 ± 0.5	7.1 ± 0.5	1.7 ± 0.5	4.0 ± 0.5	3.7 ± 0.3
Chocolate candy	4.2 ± 0.6	9.6 ± 0.7	1.3 ± 0.6	8.2 ± 0.6	1.9 ± 0.7	5.5 ± 0.6	4.3 ± 0.3
Candy, hard, skittles, starburst, *etc.*	2.3 ± 0.6	6.6 ± 0.6	2.4 ± 0.6	4.8 ± 0.6	2.4 ± 0.6	6.1 ± 0.6	4.3 ± 0.3
Snack chips like potato chips, tortilla chips, Fritos, Doritos, popcorn	6.3 ± 0.7	17.9 ± 0.8	4.5 ± 0.7	12.1 ± 0.7	5.2 ± 0.8	14.6 ± 0.7	8.7 ± 0.4
MEAT, FISH, POULTRY, MEAT SUSTITUTES							
Pizza	2.7 ± 0.4	5.2 ± 0.4	2.7 ± 0.4	4.6 ± 0.4	2.4 ± 0.4	5.5 ± 0.4	3.7 ± 0.2
Meat substitutes (veggie burgers, chicken, hot dogs or lunch meats)	1.7 ± 0.2	0.0 ± 0.2	0 ± 0.2	0.8 ± 0.2	0.5 ± 0.2	0.0 ± 0.2	0.2 ± 0.1
Hamburgers or cheese burgers	1.7 ± 0.4	9.0 ± 0.5	3.2 ± 0.4	4.6 ± 0.4	2 ± 0.5	7.9 ± 0.4	4.2 ± 0.2
Hot dogs or sausage (Polish, Italian or chorizo)	0.8 ± 0.3	3.9 ± 0.4	2.6 ± 0.4	2.0 ± 0.4	1.9 ± 0.4	5.8 ± 0.3	2.6 ± 0.2
Lunch meats (turkey or regular)	4.8 ± 0.6	9.5 ± 0.7	4.1 ± 0.6	7.8 ± 0.6	4.6 ± 0.7	9.0 ± 0.6	6.2 ± 0.3
Tacos, burritos, enchiladas, tamales with meat or chicken	2.2 ± 0.3	3.8 ± 0.3	1.8 ± 0.3	3.8 ± 0.3	1.8 ± 0.3	3.4 ± 0.3	2.7 ± 0.2
Ribs, spareribs	0.2 ± 0.1	0.5 ± 0.1	0.6 ± 0.1	0.2 ± 0.1	0.5 ± 0.1	1.2 ± 0.1	0.6 ± 0.1
Liver (chicken livers or liverwurst)	0.1 ± 0.1	0.1 ± 0.1	0.2 ± 0.1	0.0 ± 0.1	0.7 ± 0.1	0.3 ± 0.1	0.2 ± 0.1
Pig’s feet, neck bones, oxtails, tongue	0.0 ± 0.1	0.2 ± 0.1	0.4 ± 0.1	0.0 ± 0.1	0.2 ± 0.1	0.8 ± 0.1	0.2 ± 0.1
Beef or pork dishes (beef stew, pot pie, hamburger helper)	0.6 ± 0.1	1.5 ± 0.2	1.8 ± 0.2	0.8 ± 0.2	1 ± 0.2	2.7 ± 0.2	1.3 ± 0.1
Fried chicken (nuggets, wings or patties)	1.2 ± 0.5	8.2 ± 0.6	4.4 ± 0.5	3.9 ± 0.5	3.0 ± 0.6	9.5 ± 0.5	5.1 ± 0.3
Roasted or broiled chicken or turkey	5.2 ± 0.4	2.6 ± 0.5	2.1 ± 0.4	4.5 ± 0.4	5.9 ± 0.5	2.4 ± 0.4	3.9 ± 0.2
Fried fish or fish sandwich	0.3 ± 0.2	1.1 ± 0.2	1.6 ± 0.2	0.6 ± 0.2	1.2 ± 0.2	2.4 ± 0.2	1.4 ± 0.1
Fish not fried	2.5 ± 0.2	0.3 ± 0.2	0.4 ± 0.2	1.6 ± 0.2	1.7 ± 0.2	0.3 ± 0.2	0.8 ± 0.1
Peanut Butter	6.7 ± 0.5	2.4 ± 0.6	1.9 ± 0.6	8.6 ± 0.6	4.8 ± 0.6	2.0 ± 0.5	4.1 ± 0.3
Peanuts, sunflower seeds, or other nuts and seeds	5.5 ± 0.4	0.9 ± 0.5	1.5 ± 0.5	3.7 ± 0.4	4.3 ± 0.5	1.3 ± 0.4	2.3 ± 0.2
BEVERAGES							
100% orange or grapefruit juice	5.4 ± 0.8	8.3 ± 0.9	9.4 ± 0.8	8.3 ± 0.8	12.5 ± 0.9	10.5 ± 0.8	9.4 ± 0.4
Hi-C, Cranberry Juice Cocktail, Hawaiian Punch, Tang	0.8 ± 0.6	6.6 ± 0.8	5.5 ± 0.7	2.6 ± 0.7	3.3 ± 0.8	9.2 ± 0.6	4.7 ± 0.4
Kool-aid, lemonade, sports drinks, or fruit flavored drinks	3.4 ± 0.7	10.9 ± 0.9	5.8 ± 0.8	5.6 ± 0.8	3.5 ± 0.9	12.8 ± 0.7	6.7 ± 0.4
Soft drinks (Coke, Sprite, Orange) regular or diet	5.9 ± 0.8	17.4 ± 0.9	2.7 ± 0.8	12.6 ± 0.8	2.5 ± 0.9	11.0 ± 0.8	7.5 ± 0.4
Water tap or bottled	29.9 ± 0.6	23 ± 0.7	25.3 ± 0.7	28.8 ± 0.7	29 ± 0.7	21.6 ± 0.6	26.9 ± 0.3
Coffee, regular or decaffeinated	9.7 ± 0.6	1.7 ± 0.7	0.4 ± 0.7	7.4 ± 0.7	1.2 ± 0.7	0.7 ± 0.6	2.0 ± 0.3

H-P, Healthy-Processed pattern; H-S, Healthy-US Southern pattern; P-S, Processed-US Southern pattern. ^1^ All values represent means ± SE; ANOVA conducted to examine differences across the seven dietary patterns for each food item showed differences at *p* < 0.001.

### 3.3. Characteristics of Participants

**Figure 1:**
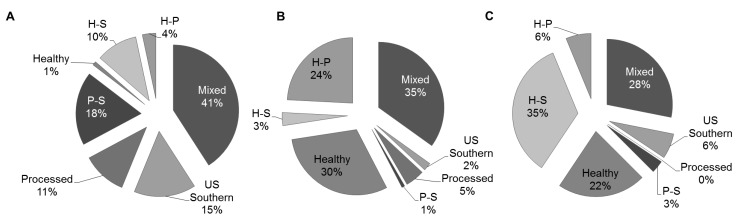
Distribution of dietary patterns during pregnancy in the Conditions Affecting Neurocognitive Development and Learning in Early Childhood (CANDLE) Cohort by race. (**a**) African American; (**b**) Caucasian; (**c**) Other.

The characteristics of the study participants according by dietary patterns appear in Table 3. Compared to the US Southern, Processed or Mixed dietary patterns women with the Healthy dietary pattern were more likely to be older (*p* < 0.0001), have a higher level of education (*p* < 0.0001), less likely to be single mothers (*p* < 0.0001), and less likely to be obese prior to pregnancy (*p* = 0.0044). The diet patterns aligned across race categories (Figure 1). Healthy and H-P patterns were consumed more by Caucasians and women in the “other” race category (Asians, American Indians, Alaska Native, Native Hawaiian, other Pacific Islander). In contrast, African Americans disproportionately were the highest consumers of the Processed, US Southern, P-S, and H-S dietary patterns. There were no significant differences in ethnicity, household size, and parity among the dietary patterns. 

The differences in mean daily macronutrient and energy adjusted mean daily micronutrient intakes among the dietary patterns appear in Table 4. Energy intake and all macro- and micronutrient intake differed among the dietary patterns (*p* < 0.001). The lowest consumption of energy adjusted nutrients was mirrored by low consumption of fruits and vegetables. The Processed and the P-S patterns had the highest energy intakes, and the Healthy and the US Southern had the lowest. The Healthy, US Southern and H-S patterns had the lowest fat and total sugar intake and highest protein intake while Processed and P-S the highest fat and sugar intake and the lowest protein intake. Regarding carbohydrate intake, there was little difference between patterns, with a significant difference found only between the Healthy and the P-S patterns (*p* = 0.011), where the Healthy group had the highest, and P-S had the lowest intake. The Healthy, H-P and H-S diets were the highest in fiber, while the Processed and P-S were the lowest. Cholesterol intake was high in the US Southern patterns and low in the Healthy and Processed patterns. The vitamin, mineral, and trace element intake was the highest in the US Southern and H-S and lowest in the Processed dietary patterns.

**Table 3:** Basic characteristics of the study participants by dietary patterns.

Variable	Healthy	Processed	US Southern	H-P	H-S	P-S	Mixed	*p* ^1^
N	135	98	120	123	98	136	445	
Age, y (mean ± SE)	30.3 ± 0.38	24.1 ± 0.49	25.2 ± 0.52	28.5 ± 4.95	27.7 ± 0.74	23.4 ± 0.40	26.2 ± 0.24	<0.0001
Height, cm (mean ± SE)	166 ± 0.64	163 ± 0.79	163 ± 0.65	164 ± 0.67	166 ± 0.74	163 ± 0.59	164 ± 0.24	0.0009
Pre-pregnancy weight, kg (mean ± SE)	68.3 ± 1.40	75.1 ± 2.35	78.1 ± 2.45	72.4 ± 1.69	77.5 ± 2.10	71.9 ± 1.82	76.2 ± 0.99	0.0005
Race, *n* (%)								
Caucasian	121 (90)	19 (19)	8 (7)	97 (79)	13 (13)	3 (2)	140 (32)	<0.001
African American	7 (5)	79 (81)	110 (92)	24 (20)	74 (76)	131 (98)	294 (66)	
Other	7 (5)	0 (0)	2 (2)	2 (2)	11 (11)	0 (0)	9 (2)	
Ethnicity, *n* (%)								
Hispanic	3 (2)	0 (0)	1 (1)	4 (3)	2 (2)	1 (1)	15 (3)	0.2424
Non-Hispanic	132 (98)	97 (100)	119 (99)	117 (97)	95 (98)	133 (99)	425 (97)	
Pre-pregnancy BMI ^2^, *n* (%)								
Underweight	7 (5)	3 (3)	5 (4)	1 (1)	3 (3)	9 (7)	19 (4)	0.0044
Normal	83 (61)	38 (39)	48 (40)	64 (52)	37 (38)	55 (40)	146 (33)	
Overweight	19 (14)	25 (25)	17 (14)	28 (23)	23 (23)	36 (26)	134 (30)	
Obese	26 (19)	32 (33)	47 (39)	30 (24)	35 (36)	36 (26)	145 (33)	
Parity, *n* (%)								
Primipara	66 (49)	44 (45)	57 (48)	49 (40)	37 (38)	47 (65)	189 (42)	0.2051
Multipara	69 (51)	54 (55)	63 (53)	74 (60)	61 (62)	89 (35)	256 (58)	
Education, *n* (%)								
Less than high school	0 (0)	16 (16)	16 (13)	3 (2)	7 (7)	29 (21)	37 (8)	<0.0001
High school or GED	23 (17)	55 (56)	62 (52)	37 (30)	41 (42)	80 (59)	216 (49)	
Technical school	8 (6)	10 (10)	12 (10)	9 (7)	11 (11)	17 (13)	49 (11)	
College or professional	102 (77)	17 (17)	30 (25)	74 (60)	39 (40)	9 (7)	143 (32)	
Marital Status, *n* (%)								
Single	5 (4)	53 (54)	71 (59)	12 (10)	39 (40)	84 (62)	160 (36)	<0.0001
Co-habitation	127 (95)	43 (44)	45 (38)	110 (90)	54 (54)	48 (35)	272 (61)	
Do not know	2 (1)	2 (2)	4 (3)	0 (0)	4 (4)	4 (3)	12 (3)	
Household size, (mean ± SE)	3.9 ± 0.1	4.7 ± 0.2	4.4 ± 1.2	4.1 ± 0.1	4.2 ± 0.2	4.6 ± 0.2	4.3 ± 0.1	0.0188
Insurance, *n* (%)								
Medicaid (Tenncare)	17 (13)	69 (70)	78 (65)	31 (25)	50 (51)	118 (87)	246 (55)	<0.0001
Other	117 (87)	28 (29)	39 (33)	92 (75)	44 (45)	17 (13)	188 (42)	
Missing	1 (0)	1 (1)	3 (2)	0 (0)	4 (4)	1 (0)	11 (3)	

H-P, Healthy-Processed pattern; H-S, Healthy-US Southern pattern; P-S, Processed-US Southern pattern. The numbers do not always add up because of rounding errors. ^1^ To test the significant differences, Kruskal-Wallis ANOVA and median test were performed. ^2^ Based on the categories established by the Institute of Medicine [25] for adults and based on age specific cut offs between age 16 and 18 years [26,27].

**Table 4:** Daily intake of total energy, macro- and energy adjusted micronutrients in the seven dietary patterns.

Nutrient	Healthy (*n* = 135)	Processed (*n* = 98)	Southern (*n* = 120)	H-P (*n* = 123)	H-S (*n* = 98)	P-S (*n* = 136)	Mixed (*n* = 445)	ANCOVA ^1^ *p*
Energy (kcal/day)	1801 ± 73.4 ^a^	2958 ± 86.2 ^b^	1887 ± 77.9 ^a^	2579 ± 76.9 ^c^	2347 ± 86.2 ^c^	3081 ± 73.2 ^b^	2360 ± 40.5 ^c^	<0.0001
Fat (% of E)	34.3 ± 0.43	37.6 ± 0.50	36.3 ± 0.46	36.6 ± 0.45	35.8 ± 0.50	37.9 ± 0.43	36.3 ± 0.24	<0.0001
Protein (% of E)	16.5 ± 0.20	13.5 ± 0.24	14.8 ± 0.21	14.9 ± 0.21	15.9 ± 0.24	14.2 ± 0.20	14.9 ± 0.11	<0.0001
Carbohydrate (% of E)	52.0 ± 0.56	50.4 ± 0.66	50.9 ± 0.59	50.5 ± 0.59	50.7 ± 0.66	49.2 ± 0.56	50.7 ± 0.31	0.0351
Saturated fat (g)	30.1 ± 0.50	33.6 ± 0.59	32.5 ± 0.53	32.2 ± 0.51	29.9 ± 0.58	34.0 ± 0.51	32.0 ± 0.27	<0.0001
Omega 3 fatty acids (g)	2.17 ± 0.05 ^b^	1.75 ± 0.06 ^c^	2.09 ± 0.06 ^b^	2.08 ± 0.05 ^b^	2.44 ± 0.06 ^a^	1.82 ± 0.05 ^c^	2.10 ± 0.03 ^b^	<0.0001
Total Sugar (g)	10.6 ± 0.64	21.7 ± 0.75	13.2 ± 0.68	16.5 ± 0.67	8.60 ± 0.75	19.4 ± 0.64	15.2 ± 0.35	<0.0001
Fiber (g)	27.4 ± 0.48	15.4 ± 0.55	20.8 ± 0.50	22.3 ± 0.49	26.0 ± 0.54	15.2 ± 0.48	20.5 ± 0.26	<0.0001
Fe (mg)	19.2 ± 0.32 ^a^^,b^	15.6 ± 0.37 ^d^	17.6 ± 0.34 ^c^	17.9 ± 0.33 ^b^^,c^	19.5 ± 0.37 ^a^	16.1 ± 0.32 ^d^	17.7 ± 0.17 ^c^	<0.0001
Zn (mg)	14.3 ± 0.23	12.2 ± 0.26	12.9 ± 0.24	13.3 ± 0.23	13.5 ± 0.26	12.5 ± 0.23	13.2 ± 0.12	<0.0001
Vit B6 (mg)	2.69 ± 0.05 ^a^	1.93 ± 0.06 ^c^	2.37 ± 0.05 ^b^	2.40 ± 0.05 ^b^	2.72 ± 0.06 ^a^	2.03 ± 0.05 ^c^	2.33 ± 0.03 ^b^	<0.0001
Vit B12 (μg)	6.54 ± 0.20 ^ab^	5.00 ± 0.23 ^d^	6.01 ± 0.20 ^a^^,b^^,c^	5.64 ± 0.20 ^c^^,d^	6.69 ± 0.22 ^a^	5.76 ± 0.20 ^b^^,c^^,d^	6.03 ± 0.10 ^a^^,b^^,c^	<0.0001
Folate (μg)	777 ± 17.0	544 ± 19.7	656 ± 17.9	715 ± 17.4	775 ± 19.4	559 ± 17.1	671 ± 9.11	<0.0001
Thiamine (mg)	2.09 ± 0.03 ^a^	1.62 ± 0.04 ^d^	1.95 ± 0.03 ^b^	1.89 ± 0.03 ^b^^,c^	2.10 ± 0.04 ^a^	1.76 ± 0.03 ^c^^,d^	1.91 ± 0.02 ^b^	<0.0001
Niacin (mg)	27.4 ± 0.45 ^a^	23.0 ± 0.53 ^d^	24.7 ± 0.48 ^c^^,d^	25.8 ± 0.46 ^a^^,b^^,c^	27.1 ± 0.52 ^a^^,b^	24.0 ± 0.46 ^c^^,d^	25.5 ± 0.24 ^b^^,c^	<0.0001
Riboflavin (mg)	2.77 ± 0.04 ^a^	2.00 ± 0.05 ^d^	2.40 ± 0.05 ^c^	2.46 ± 0.04 ^b^^,c^	2.65 ± 0.05 ^a^^,b^	2.11 ± 0.04 ^d^	2.42 ± 0.02 ^c^	<0.0001
Total Choline (mg)	386 ± 7.08 ^b^	272 ± 8.22 ^f^	377 ± 7.44 ^b^^,c^	334 ± 7.23 ^d^^,e^	435 ± 8.08 ^a^	328 ± 7.11 ^e^	354 ± 3.79 ^c^^,d^	<0.0001
Free Choline (mg)	98.3 ± 1.33 ^a^	63.8 ± 1.54 ^d^	79.5 ± 1.39 ^c^	88.2 ± 1.35 ^b^	94.0 ± 1.51 ^a^^,b^	65.9 ± 1.33 ^d^	81.9 ± 0.71 ^c^	<0.0001

H-P, Healthy-Processed pattern; H-S, Healthy-US Southern pattern; P-S, Processed-US Southern pattern. Results are given as mean ± SE. ^1^ Covariate: Energy intake (kcal/day). ^a^^,b^^,c^^,d^^,e^^,f^ Means that do not share the same superscript are significantly different from each other.

## 4. Discussion

Three major dietary patterns, namely Healthy, Processed and US Southern were identified among pregnant women from the Mid-South using factor analysis. Combining the factor scores with quintile rankings of the factors we developed seven distinct mutually exclusive dietary patterns. This is the first study that examined dietary patterns within a geographical region that includes a diverse socioeconomic sample from the US. These dietary patterns were different (*p* < 0.001) from each other in almost all the food items, macro- and micro nutrients and aligned across socioeconomic and racial groups. 

Dietary patterns are known to vary with age, gender, economics, and cultural habits [28]. Residents of Shelby County Tennessee reflect a diverse population that includes a preponderance of African-Americans across a wide range of incomes. The CANDLE study has enrolled participants reflective of the birth mothers of Shelby County Tennessee, thus diet characterization must be sensitive to the different segments of the population. Our approach to identifying dietary patterns appears to have been successful in identifying various segments of our study population based on race, income, and education. While this report is not unique with regard to dietary patterns [15] as they relate to segments of the population, it did identify types and combinations of foods that have distinct differences in nutrient composition with regard to nutrients linked to neurocognitive development [18,29].

Our findings that healthy eating patterns are reflective of older and more educated individuals are in concert with other reports [14]. Our Southern diet pattern is reflective of foods traditionally ascribed to the southeastern US [30]. The identified patterns in this paper are comparable and representative of the Southern US regions that differ from the national patterns [31,32,33]. Substantial segments of our sample retained core southern foods while also incorporating items from the healthy and from the processed foods patterns, reflecting a wider variety of food selection that translated into different nutrient intakes. Those with the H-S dietary pattern had higher levels of omega-3 fatty acids, iron, vitamin B_6_, folate, thiamine, niacin, riboflavin, total choline, and free choline than those with the pure Southern diet pattern, implying that individuals in this category capitalized on the foods with the highest nutrient density of both eating patterns. The Processed dietary pattern was characterized by a lower nutrient density and high energy content, yielding a decreased energy adjusted nutrient intake when combined with other dietary patterns. This is consistent with diets associated with food globalization, urbanization, and lower economic status [34]. The diversity of food selection among the African-American and Caucasian participants in our study underscores cultural sensitivity at the local level is important when collecting dietary information [30].

One limitation of our study was the significant amount of missing data due to technical problems and too low or high energy intake. The excluded sample consisted mainly of African American women due to the study design at recruitment. Therefore the missing sample is not representative of the overall study population presented in this paper, but after stratification, it is representative of the African American study population. Since our results show racial difference in the distribution of study participants across the dietary patterns, we think that this limitation has no significant effect on our analysis. 

Statistical approaches have been well documented in establishing dietary patterns. Depicting dietary patterns using factor analyses can account for 15%–32% of the variance in dietary intake [7,15,35,36]. Explained variance in factor analysis is influenced by the amount of variables that are used in the analysis. Our result of the 15.4% is comparable with previously published literature in Mexican Americans [37], however they used 63 food items to identify dietary patterns which means potentially less variance, as the 111 items in our analysis. Most approaches are based on a 1 or 2 step process that may or may not allow for accurate calculation of total variance. However, the dietary patterns in our study accounted for 89%–90% of the variance in energy adjusted macronutrients and 50%–78% of the variance in the energy adjusted micronutrients. Thus, our patterns may be more robust in examining associations with cognitive development than single nutrients alone.

The complexity of a two-step process in assigning dietary patterns allowed us to capture mixed patterns. An individual with a high score on one factor may have another high score on another factor; therefore, the person’s dietary pattern is a mixed pattern and not a pure dietary pattern of the highest score. The interpretation of these cases is analytically challenging because there are no cutoffs for these cases. Investigators using factor analysis should be cautious about this problem. To overcome this problem, we created mixed dietary patterns based on the individuals’ rank orders in each factor. In this way H-P, H-S, P-S, and overall Mixed patterns were identified. The analysis of the individual food items and energy, macronutrient, and micronutrient intake of these patterns confirm that they are significantly distinct from the other main food patterns. Knudsen and colleagues [16] have used this method of analysis where they identified two factors, *i.e.*, healthy and unhealthy patterns, and then created a third, mixed dietary pattern based on quintiles of the factor scores. Two thirds of their study population belonged to this intermediate group, which is consistent with our results. We found that 70% of the participants in our study ate foods consistent with the mixed dietary pattern.

Our statistical approach was to use all 111 food groups compared to condensing the food items into smaller groups. While decreasing the number of groups may increase the variance explained by the factor analysis, it decreases the ability to identify unique dietary patterns within sub groups of the population perspective [38]. For example condensing food items to 46 groups may yield two-three factors and thus limits the description of the dietary patterns. We also examined if incorporating the fat content of food item yielded additional information pertinent to dietary patterns. In our sample, this did not yield any additional information.

The goal of determining diet patterns is often to identify combinations of foods and beverages that reflect a specific type of diet that may be beneficial or harmful to a health outcome. The strengths and limitations of classifying individuals into specific patterns are that they may reflect an overall pattern of intake that may be targeted for community interventions; however, this may not be useful in examining a specific nutrient/disease relationship. Use of the seven patterns may not be readily translated into individual diet counseling, but it does identify key foods that could be targeted for community-based interventions to improve nutrient intake. Selectively over or under reporting by individuals of various socio-demographic groups, may bias the results of a study using nutrients as the exposure [39]. Diet patterns appear to be unaffected by under reporting or the report of consuming a food item [40]. Our approach in classifying diets, clearly links consumption of more processed foods (low nutrient density) with a higher percentages of overweight and obesity, thereby suggesting that under reporting may not be a large factor in this study. 

## 5. Conclusions

Our study is the first to provide unique dietary patterns consumed by a cohort of women living in the mid-southern US. The diet patterns reflect a gamut of food stuffs that describe the traditional southern diet in the US, a highly processed diet, a primarily healthy food and dietary pattern combinations that reflect nutrition transition. The goal of creating diet patterns for this prospective study is to use examine the association of maternal nutritional factors during pregnancy to brain and cognitive development by age 3. How the dietary patterns during pregnancy relate to the child’s cognitive development or help explain epigenetic expression of disease or health condition remains to be determined. In our longitudinal study, we will investigate whether maternal nutrient intake and diet patterns during pregnancy are somewhat stable and examine the influence on early childhood development as part of the life course history.

## Supplementary Files

Supplementary File 1 Supplementary Information (PDF, 102 KB)
