# Supplementary material for: Dietary Patterns in Pregnancy and Effects on Nutrient Intake in the Mid-South: The Conditions Affecting Neurocognitive Development and Learning in Early Childhood (CANDLE) Study
Source: Nutrients. 2013 May 3;5(5):1511–30. doi: 10.3390/nu5051511 (PMC3708333; doi:10.3390/nu5051511)
Supplement: Supplementary File 1 — Supplementary Information (PDF, 102 KB) [file nutrients-05-01511-s001.pdf]

Supplementary Information

Table S1. Factor loadings of all food and beverage items/groups in the three main factors.

Food item	Healthy	Processed	US Southern
**(variance explained)**	**−5.80%**	**−5.10%**	**−4.40%**
Eggs	0.090	0.031	0.344
Breakfast sausage including in sandwiches/biscuits	−0.190	0.301	0.311
Bacon	−0.118	0.148	0.367
Pancakes, waffles, French toast or Pop tarts	−0.030	0.249	0.205
Cooked cereals (oatmeal, grits, cream of wheat)	0.157	−0.095	0.320
Breakfast or cereal bars	0.079	0.141	0.098
Milk or milk substitutes on cereal	0.067	0.148	0.123
Yogurt, including frozen	0.434	−0.115	0.059
Cheese, sliced or spreads	0.246	0.332	−0.095
Banana	0.334	−0.025	0.201
Apples or pears	0.355	−0.056	0.277
Oranges or tangerines	0.276	−0.077	0.281
Grapefruit	0.243	−0.091	0.125
Peaches or nectarines, fresh	0.135	−0.082	0.352
Other fresh fruits like grapes, plums, honeydew, mango	0.248	0.017	0.290
Canned fruit	0.034	0.129	0.374
Cantaloupe in season	0.187	−0.005	0.147
Strawberries or other berries in season	0.383	-0.056	0.135
Watermelon in season	0.095	−0.028	0.205
Broccoli	0.331	−0.059	0.312
Carrots or mixed vegetables with carrots	0.471	−0.026	0.124
Corn	0.203	0.134	0.316
Green beans or green peas	0.292	0.043	0.407
Spinach, cooked	0.370	−0.115	0.106
Greens (collards, turnip, or mustard)	0.074	0.033	0.517
Sweet potatoes, yams	0.164	−0.080	0.360
Fried potatoes (French fries, home fries, hash browns)	−0.125	0.563	0.111
Potatoes not fried (mashed, boiled, baked, or potato salad)	0.270	0.191	0.138
Cole slaw, cabbage, Chinese cabbage	0.194	−0.028	0.444
Green salad, lettuce salad	0.600	−0.052	0.015
Tomatoes, raw	0.524	0.005	−0.060
Salad dressing, regular or low fat	0.541	0.035	0.072
Other vegetables (squash, cauliflower, okra, peppers)	0.547	−0.152	0.005
Refried beans or bean burritos	0.269	0.098	−0.073
Pinto, black or baked beans, chili with beans	0.304	0.097	0.012
Vegetable stew (without meat)	0.227	−0.121	0.200
Vegetable, vegetable-beef or tomato soup	0.337	−0.042	0.200
Split pea, bean or lentil soup	0.163	0.018	0.021
Any other soup (chicken noodle, cream sous, Cup-A-Soup, ramen	0.004	0.281	0.207
Pizza	−0.033	0.328	0.055
Spaghetti, lasagna or any other pasta with tomato sauce	0.217	0.203	0.123
Macaroni and cheese	−0.001	0.255	0.247
Other noodles (egg noodles, pasta salad, sopa seca)	0.176	0.037	0.072

Table S1. *Cont.*

Tofu or tempeh	0.249	−0.080	−0.119
Meat substitutes (veggie burgers, chicken, hot dogs or lunch meats)	0.346	−0.060	−0.093
Hamburgers or cheese burgers	−0.227	0.504	0.073
Hot dogs or sausage (Polish, Italian or chorizo)	−0.134	0.342	0.271
Lunch meats (turkey or regular)	0.082	0.393	0.101
Meat loaf, meat balls	0.032	0.135	0.242
Steak, roast beef, or beef in frozen dinners or sandwiches	0.158	0.188	0.052
Tacos, burritos, enchiladas, tamales with meat or chicken	0.026	0.306	-0.008
Ribs, spareribs	−0.060	0.164	0.373
Pork chops, pork roasts, cooked ham	−0.112	0.179	0.268
Veal, lamb, deer meat	0.146	−0.013	0.006
Liver (chicken livers or liverwurst)	0.091	−0.002	0.319
Pigs feet, neck bones, oxtails, tongue	−0.128	0.121	0.419
Menudo, pozole, caldo de res, sanchoco, ajiaco	0.034	0.022	−0.021
Beef or pork dishes (beef stew, pot pie, hamburger helper)	−0.092	0.220	0.319
Fried chicken (nuggets, wings or patties)	−0.163	0.497	0.274
Roasted or broiled chicken or turkey	0.354	−0.024	0.059
Any other chicken dish (chicken stew, chicken noodles, chicken salad, Chinese chicken dishes)	0.291	0.045	0.014
Oysters	0.141	−0.055	−0.029
Shellfish (shrimp, scallops, crabs)	0.152	0.042	0.163
Tuna, tuna salad, tuna casserole	0.184	0.041	0.262
Fried fish or fish sandwich	−0.075	0.178	0.378
Fish not fried	0.435	−0.099	−0.031
Biscuits, muffins, croissants (without eggs)	0.152	0.251	0.163
Sandwich buns	0.032	0.469	0.000
Bagels, English muffins, dinner rolls	0.339	0.094	−0.020
Tortillas (don't include tacos or burritos)	0.167	0.047	−0.056
Cornbread, corn muffins, hush puppies	0.010	0.286	0.380
Sliced bread (white, dark, whole wheat)	0.264	0.310	−0.018
Rice or rice dishes	0.291	0.077	0.166
Margarin (not butter) on bread or vegetables	0.067	0.249	0.003
Butter (not margarine) on bread or vegetables	0.082	0.261	0.088
Energy bars, like Power bars, Clif bars, Balance, Luna	0.186	0.015	−0.055
Breakfast or cereal bars	0.308	0.020	0.019
Peanuts, sunflower seeds, or other nuts and seeds	0.348	−0.049	0.034
Peanut Butter	0.378	0.043	−0.115
Snack chips like potato chips, tortilla chips, Fritos, Doritos, popcorn	-0.077	0.552	0.022
Crackers (Saltines, Cheez-It, or any other snack cracker)	0.236	0.250	0.096
Jelly, jam	0.156	0.209	0.086
Mayonnaise, sandwich breads	0.012	0.372	0.097
Ketchup, salsa or chili peppers	0.050	0.450	−0.084
Mustard, barbecue sauce, soy sauce, gravy, *etc.*	0.200	0.322	−0.039
Donuts	0.043	0.304	0.019
Cake, snack cakes, cupcakes, Ho-Hos, pastries	-0.057	0.437	−0.017
Cookies	0.182	0.407	−0.086

Table S1. *Cont.*

Ice cream, ice cream bars	0.106	0.226	0.115
Chocolate syrup or sauce (in milk or ice cream)	0.126	0.084	−0.152
Pumpkin pie, sweet potato pie	0.041	0.072	0.213
Other pies (apple pie, cherry pie, fast food pies, *etc.*)	0.052	0.285	0.032
Chocolate candy	0.091	0.392	−0.130
Candy, hard, skittles, starburst, *etc.*	−0.014	0.358	0.073
Milk as a beverage	0.303	−0.044	−0.024
Drinks like Slim Fast, Sego, Slender, Ensure or Atkins	−0.028	−0.018	0.027
Tomato juice or V-8 juice	0.241	0.012	0.080
Apple juice, grape juice, pineapple juice or fruit smoothies	0.234	0.095	0.170
100% orange or grapefruit juice	0.145	0.133	0.310
Hi-C, Cranberry Juice Cocktail, Hawaiian Punch, Tang	−0.132	0.309	0.274
Drinks with some juice (Knudsen, Sunny Delight)	−0.161	0.210	0.247
Ice tea (homemade, instant, bottled)	0.074	0.154	−0.083
Kool-aid, lemonade, sports drinks, or fruit flavored drinks	−0.145	0.356	0.136
Soft drinks (Coke, Sprite, Orange) regular or diet	−0.115	0.377	−0.227
Beer or non-alcoholic beer	0.147	0.054	−0.160
Wine or wine coolers	0.184	−0.014	−0.182
Liquor or mixed drinks	−0.059	0.022	0.009
Water tap or bottled	0.320	−0.111	−0.059
Coffee (regular or decaf)	0.314	−0.013	−0.282
Hot tea (not including herbal teas)	0.218	−0.019	−0.166

© 2013 by the authors; licensee MDPI, Basel, Switzerland. This article is an open access article distributed under the terms and conditions of the Creative Commons Attribution license (http://creativecommons.org/licenses/by/3.0/).
